# Decontamination efficiency of sodium-bicarbonate and glycine air-polishing agents on sand-blasted acid-etched titanium surface and its toxicity against human gingival fibroblasts

**DOI:** 10.1016/j.jobcr.2026.101550

**Published:** 2026-09-22

**Authors:** R. Muthu Mekala, Santhosh Kumar, B. Rajasekaran, S. Pradeep, Sangeeta Umesh Nayak

**Affiliations:** a Department of Periodontology, Manipal College of Dental Sciences, Manipal Academy of Higher Education, Manipal, Udupi, Karnataka, India; b Department of Metallurgical & Materials Engineering, National Institute of Technology, Surathkal, Karnataka, India; c Department of Prosthodontics and Crown and Bridge, Manipal College of Dental Sciences, Manipal Academy of Higher Education, Manipal, Udupi, Karnataka, India; d Department of Periodontology, Manipal College of Dental Sciences, Mangalore, Manipal Academy of Higher Education, Manipal, Karnataka, India

**Keywords:** Biocompatible materials, Decontamination, Fibroblasts, Glycine, Sodium bicarbonate, Titanium

## Abstract

**Objectives:**

To evaluate and compare glycine's decontamination efficiency and biocompatibility with sodium bicarbonate airprophy agent.

**Materials and methods:**

Forty-two sites on round titanium discs were subjected to sandblasting and a double acid etching. Discs were then divided into group 1 and group 2 after applying artificial calculus. They were treated using sodium bicarbonate or glycine airprophy agents. A profilometer was used to analyze the roughness, and SEM was used to assess the morphology. Cytotoxicity to human gingival fibroblasts of five treated discs from each group was evaluated by MTT assay.

**Results:**

After decontamination, the median Ra values of Group 1 and Group 2 discs were 1.51 μm and 1.43 μm respectively, before the treatment, and after the treatment, they were 1.96 μm and 1.83 μm respectively. Similarly, the median Rz values increased from 9.27 μm and 9.30 μm before treatment to 13.18 μm and 13.26 μm after treatment in groups 1 and 2, respectively. However comparison between the groups using the Mann-Whitney U test revealed no statistically significant differences (p > 0.05). The MTT assay showed that there was 80% cell viability following exposure to sodium bicarbonate and glycine, indicating acceptable cytocompatibility under the conditions of the study.

**Conclusion:**

Glycine and sodium bicarbonate are equally effective in debriding artificial calculus and are non-toxic.

## 1 Introduction

Professional maintenance and following appropriate oral hygiene measures is very important to preserve the soft and hard tissues around the implant.^1^ In patients with peri-implantitis, the bone level is reduced that leads to reduced function. To promote re-osseointegration, peri-implant bone regeneration needs to be performed.^2^ During this procedure, surface decontamination restores the health of the implant surface. The methods employed to clean the implant surface ideally need to be tissue friendly and should be effective in removing adhered dental plaque and embedded calculus.^3^

Metal instruments used for debridement cause surface alterations on the collar and the threads of the implants. The roughness created enhances bacterial growth and causes plaque formation, and thus affecting tissue adhesion.^2^ Hence, care should be taken not to damage the implant surface coating or cause alterations to the implant surface roughness. Thus, to maintain implant surface integrity, non-metal instruments or air polishing devices can be considered. It can reduce the damage created on the dental implant surface, thus improving the soft tissue attachment. Metallic instruments need maintenance to enhance their efficiency and durability and need constant replacement over time. Studies have shown that it causes damage to the surface of titanium and can produce only soft tissue attachment rather than bone regeneration during periodontal healing.^4^ Concerning air prophy devices, the polishing powder needs to be replaced, and the handpiece needs regular inspection. Among the air abrasive agents' sodium bicarbonate is commonly used for implant surface decontamination due to its larger particle size. But it is abrasive to the implant surface and can produce soft tissue trauma. To minimize tissue damage and to avoid implant surface alterations, less abrasive air polishing powders like glycine have been used, which have a smaller particle size compared to sodium bicarbonate. Many studies have tested glycine's efficiency in the removal of artificial biofilm.^5^ Studies evaluating the efficiency of the removal of artificial calculus are scarce.^2^

The present study was conducted to evaluate the efficiency of glycine air abrasive in the removal of artificial calculus from the titanium discs that simulated the dental implant surface. This was compared with sodium bicarbonate air abrasive powder. The parameters explored were the surface alteration produced by the air abrasive powder and its efficiency in the removal of the artificial dental calculus, as well as to assessing biocompatibility by checking human gingival fibroblast cell viability to those air abrasive powders. It was hypothesized that glycine air abrasive produces less surface alteration while decontaminating hardened deposits and is more biocompatible than sodium bicarbonate air abrasive.

## 2 Materials and Methods

The Present study was done in the department of Periodontology. Ethical approval was obtained from Kasturba hospital (KMC and KH). The ethical clearance number is IEC:808/2020. Based on the previous study ^2^ sample size was estimated using the standard deviation and the anticipated effect size. Assuming a two sided significance level of 5%, a statistical power of 80%, and a normal distribution of the outcome variable, a minimum sample size of 14 samples was required. The samples were equally distributed between control and test groups (n = 7 per group) to evaluate differences in decontamination efficiency.

## 3 Materials

14 round titanium discs (Grade 5) of 10 mm diameter and 3 mm thickness were procured from Bhagyasali metals, Mumbai. Silicon carbide powder for sandblasting on titanium discs was bought from Sarrah and Sons company. Artificial dental calculus set was bought from Nissin Dental Products company, Kyoto, Japan. Sodium bicarbonate was provided by Galaxy Air Polishers and glycine was provided by Gene Technologies, Bio-Rad, Chennai.

The powders used were glycine powder with granulometry of 65 μm and sodium bicarbonate powder with a granulometry of 150 μm. To standardize air-polishing treatment, each titanium disc was kept on a custom-made acrylic resin block during air prophy treatment (Fig. 1).

**Fig. 1:**
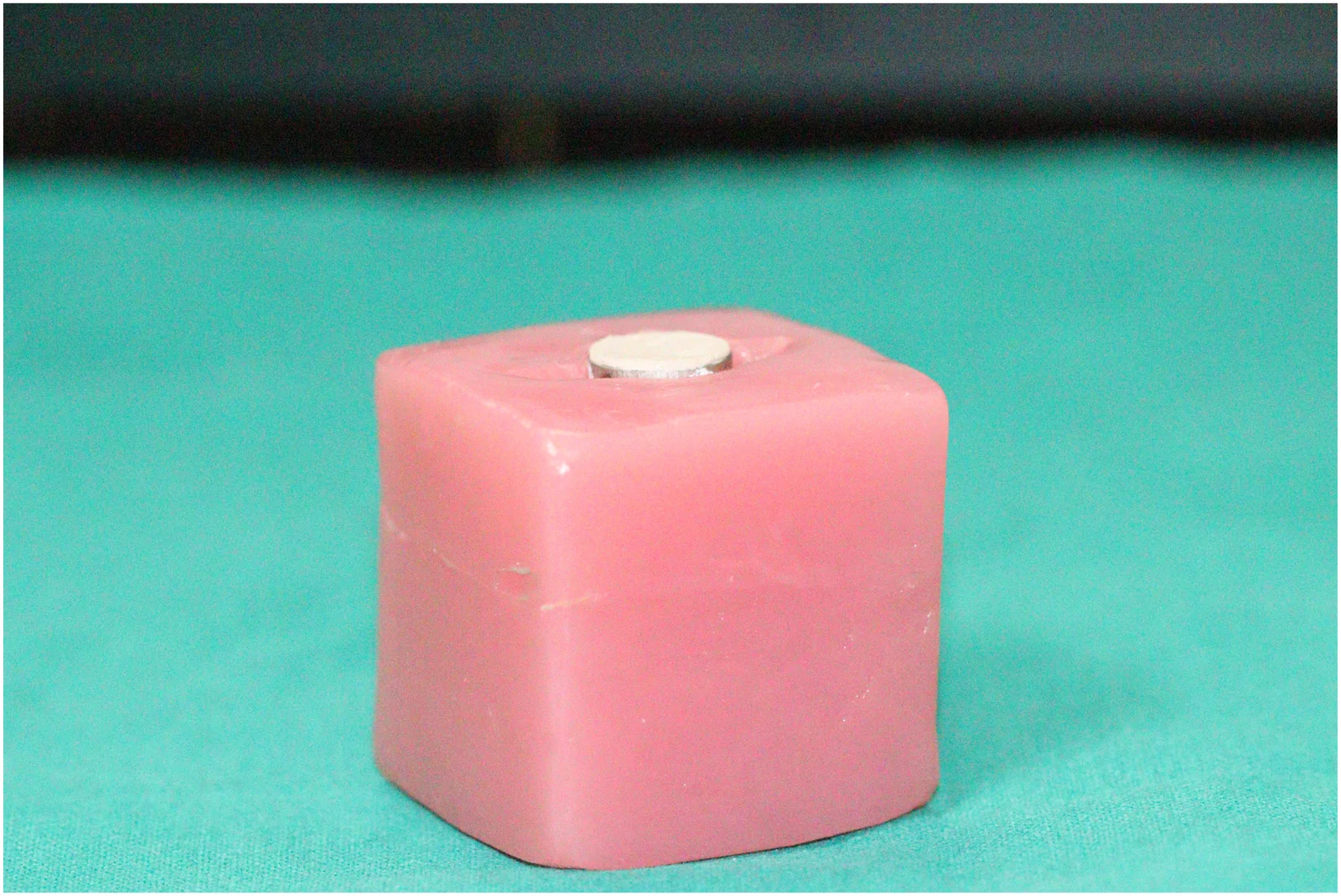
Titanium disc mounted on an acrylic base.

To simulate dental implant, surface the discs were treated by sand blasting and acid-etching process (Department of Chemistry, MIT, Manipal).

The cell line used for the study was Human gingival fibroblasts (HGFs) from the repository of Central research laboratory (Maratha Mandal Dental college).

## 4 Methodology

### 4.1 Sandblasting

For Sandblasting, silicon carbide particles are used to blast and create craters on the surface of titanium. The nozzle (Fig. 2) was kept at a distance of 2 cm from the disc at a 90° angle and the pressure was set at 55 psi. The sandblasting was done for 30 s.

**Fig. 2:**
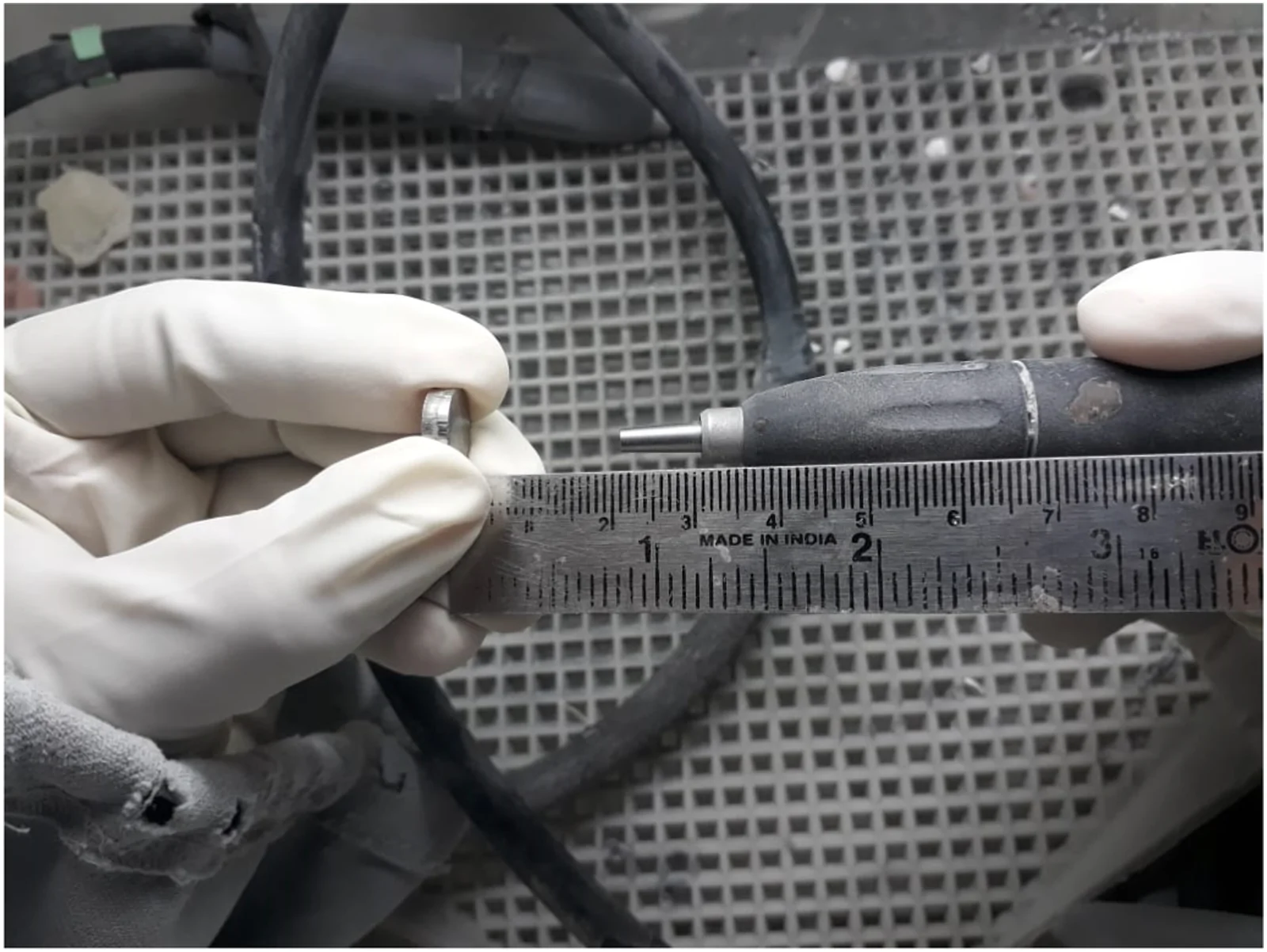
Sandblasting of the titanium discs.

### 4.2 Acid etching

Following the sandblasting, the implant discs were subjected to a double acid etching process using 49% H_2_SO_4_ mixed with 18% HCL for 30 min at elevated temperature >100^o^ C to remove remnant blasting particles embedded on the surface and to further alter the surface topography and chemistry.

### 4.3 Application of artificial dental calculus

Artificial dental calculus (ADC) was mixed as per the manufacturer's instructions, and a uniform thickness of 2 mm (Fig. 3a) was applied to the discs. Once the material set and hardened on the surface, a portion of the bulk layer covering the disc surface was removed using a blunt plastic instrument without damaging the disc surface. A uniform layer of artificial calculus of less than 1 mm was observed, and the calculus got embedded into the micro-roughened disc surface (Fig. 3b). The discs were then placed on an acrylic base, and the air powder abrasive system containing sodium bicarbonate powder was used to remove the ADC deposits on the surface in group 1, and the glycine powder air abrasive system is used in group 2.

**Fig. 3:**
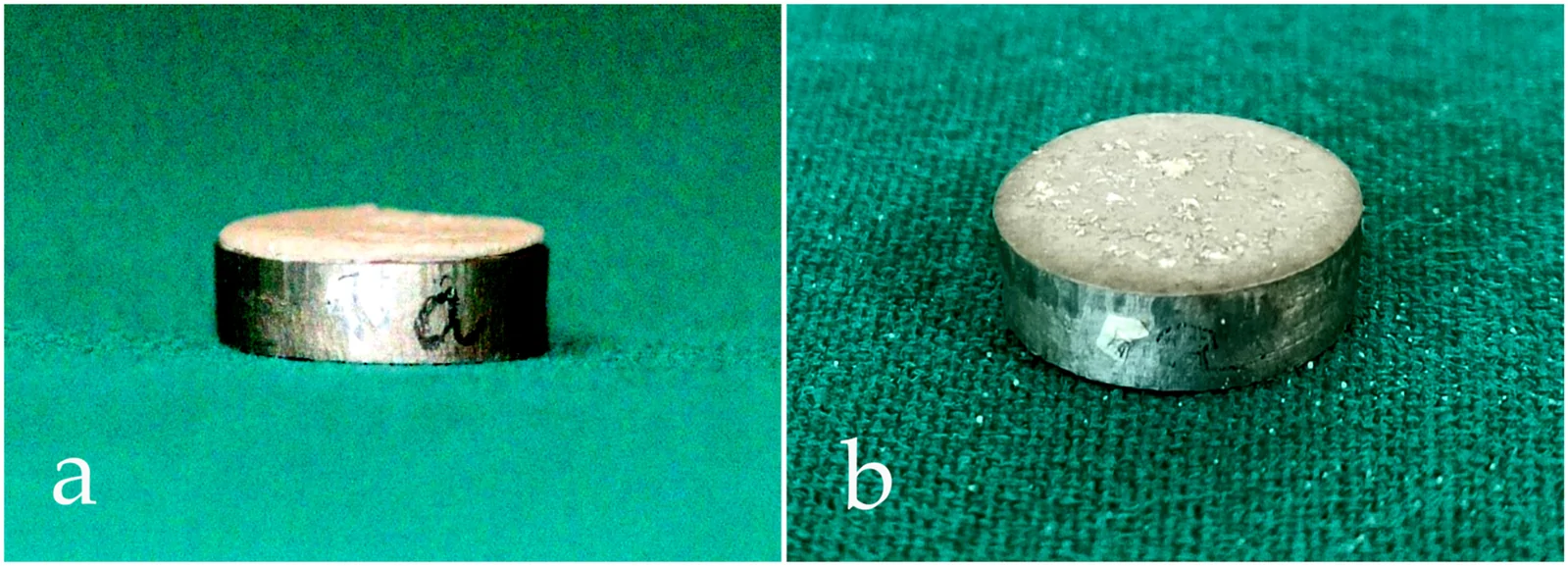
Application of artificial dental calculus.

### 4.4 Air polishing

The jet of polishing powder using the air prophy unit was applied with the nozzle kept at an average distance of 2 to 3 cm at an angle of 45° relative to the discs. The pressure was maintained at 80 pounds per square inch (psi) in accordance with the study by Gehrke et al. for both materials until the artificial calculus was completely removed (by visual inspection).^2^

### 4.5 Roughness analysis (Ra and Rz values)

Ra is the arithmetic average of heights measured across a surface. Simply averaging the height across the microscopic peaks and valleys. Rz is the average value of the absolute values of the heights of the five highest-profile peaks and the depths of the five deepest valleys within the evaluation length.

Surface roughness analysis was done using a stylus profilometer (Surtronic 3+ Taylor Hobson) before and after the treatment. Each disc was divided into 3 surfaces on the top, where SLA treatment was done, named as (surface a, surface b & surface c) (Fig. 4), and the roughness measurements (both Ra & Rz) were taken from these 3 surfaces on each titanium disc for the analysis (total 42 sites). Group matching was done using the Ra values, and the discs were randomly assigned to group 1 and group 2, consisting of 7 discs in each.

**Fig. 4:**
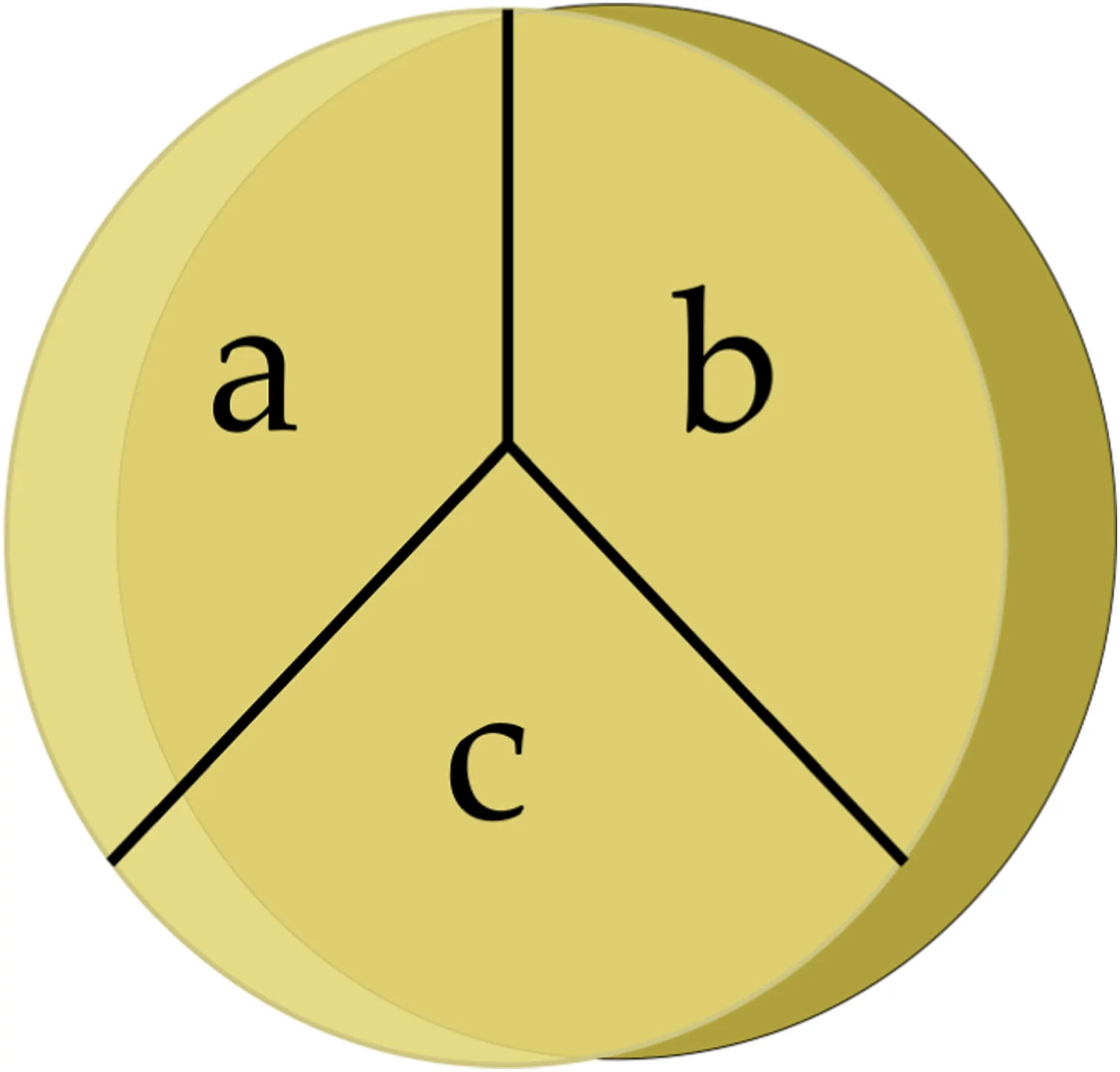
Division of the regions on the surface of the discs for evaluation of the roughness and the morphology.

### 4.6 Scanning electron microscopy analysis

Evaluations of surface topography were made on 14 decontaminated titanium discs using images captured on a scanning electron microscope (EVO MA18 with Oxford EDS (X-act) at magnifications of 750x, 1000x, and 1500x before and after the application of artificial calculus (Fig. 5a & b). The completeness of calculus removal was also evaluated at these magnifications (Fig. 6a and b). Energy-dispersive X-ray analysis was done to find out elemental composition of the treated titanium surface. Surface roughness analysis was repeated after the treatment of the discs using the air prophy unit.

**Fig. 5:**
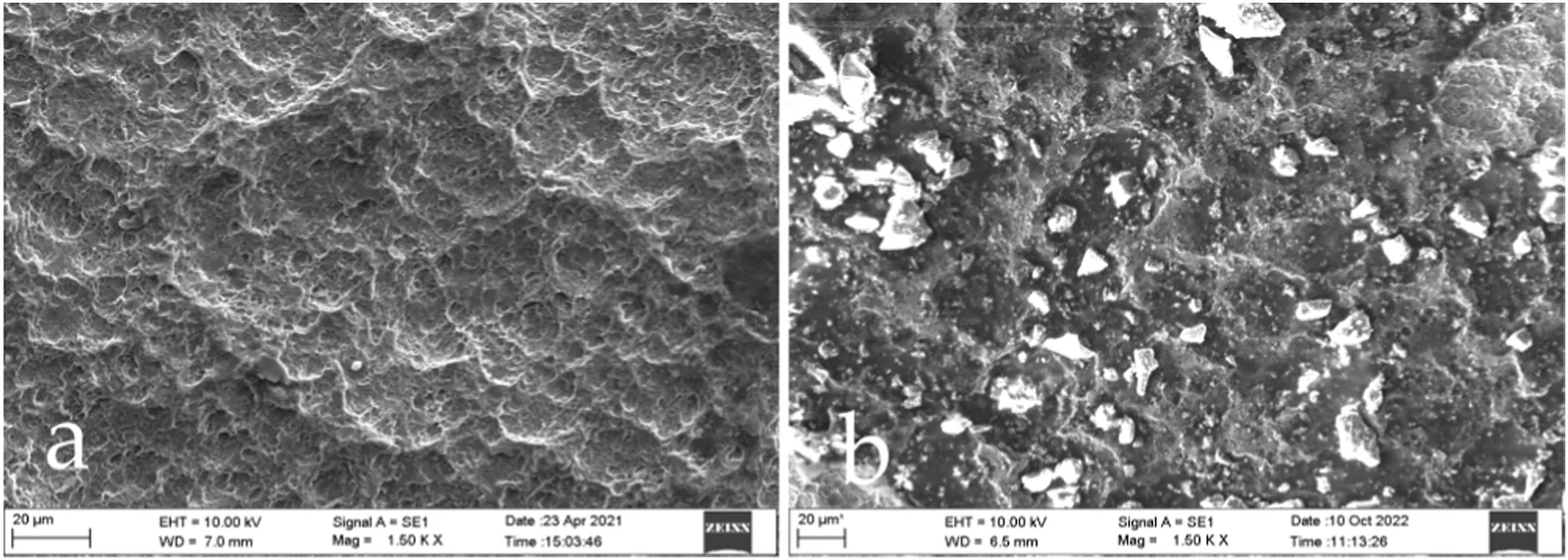
Pre (a) and Post (b) application of the artificial dental calculus on the surface of the titanium disc.

**Fig. 6:**
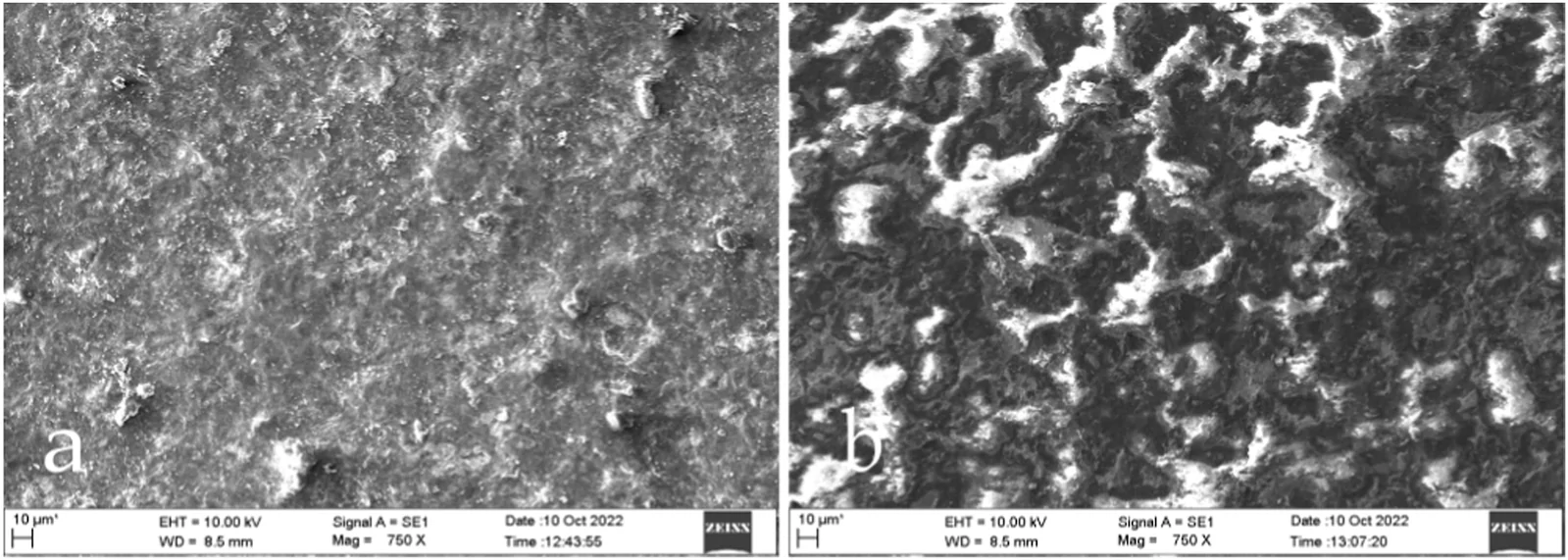
a: Decontamination using the sodium bicarbonate and b: decontamination using the glycine air polishing agent.

### 4.7 MTT assay

Cell culture:

Human gingival fibroblasts (HGFs) cell lines were maintained in 96 wells micro titer plate containing MEM media supplemented with 10% heat-inactivated fetal calf serum (FCS), containing a 5% mixture of Gentamicin (10 μg/ml), Penicillin (100 μg/ml), and Streptomycin (100 μg/ml) in the presence of 5% CO_2_ at 37°C for 48-72 h.

Cytotoxicity Assay:

In vitro growth inhibition effect of the test compound was assessed by calorimetric or spectrophotometric determination of conversion of MTT into “Formazan blue” by living cells.

a) The supernatant from the plate was removed, and fresh MEM solution was added. The discs were placed maintaining complete sterility. The control group contained only DMEM.
b) After 72 hours of incubation at 37°C in a humidified atmosphere of 5% CO_2_, stock solution of MTT was added to each well (20 μl, 5 mg per ml in sterile PBS) for a further 4 h incubation.
c) The supernatant was carefully aspirated, the precipitated crystals of “Formazan blue” were solubilized by adding DMSO (100 μl), and optical density (OD) was measured at a wavelength of 570 nm using a LISA plus (ELISA reader).

Formula:

$$Surviving\;cells\left(\%\right)\frac{Mean\;OD\;of\;test\;compound}{Mean\;OD\;at\;control}X100$$

## 5 Statistical analysis

Data was analyzed using the statistical package SPSS 26.0 (SPSS Inc., Chicago, IL) and level of significance was set at p < 0.05. Inferential statistics to determine the difference between groups were done using Mann Whitney *U* test and within the groups using Wilcoxon signed-rank test.

## 6 Results

### 6.1 Comparison within the group (Wilcoxon signed-rank test)

The median Ra values of 3 surfaces (a, b, c) of group 1 (sodium bicarbonate treated) discs before the treatment were 1.51 (1.42 to 1.89 μm). After the treatment with the abrasive agent, the value was 1.96 (1.86 to 2.50). Significant differences were found between before/after values (Table 1), p < 0.05. The median Rz value of 3 surfaces (a, b, c) of group 1 (sodium bicarbonate treated) discs was 9.27 (8.85 to 11.5) (Fig. 7). After the treatment, the value was 13.18 (12.4 to 16.01). There was a significant difference found between before/after values (Table 1), p < 0.05.

**Table 1:** Group descriptive of the roughness values of sodium bicarbonate and glycine.

Air polishing powders					Percentiles		
		N	Median	IQR	25th	50th	75th
NaHC (Ra) μm	Before	21	1.51	0.470	1.42	1.51	1.89
	After	21	1.96	0.640	1.86	1.96	2.50
NaHC (Rz) μm	Before	21	9.27	2.650	8.85	9.27	11.50
	After	21	13.18	3.610	12.40	13.18	16.01
Gly (Ra) μm	Before	21	1.43	0.180	1.40	1.43	1.58
	After	21	1.83	0.880	1.45	1.83	2.33
Gly (Rz) μm	Before	21	9.30	0.980	8.83	9.30	9.81
	After	21	13.26	6.080	10.64	13.26	16.72

**Fig. 7:**
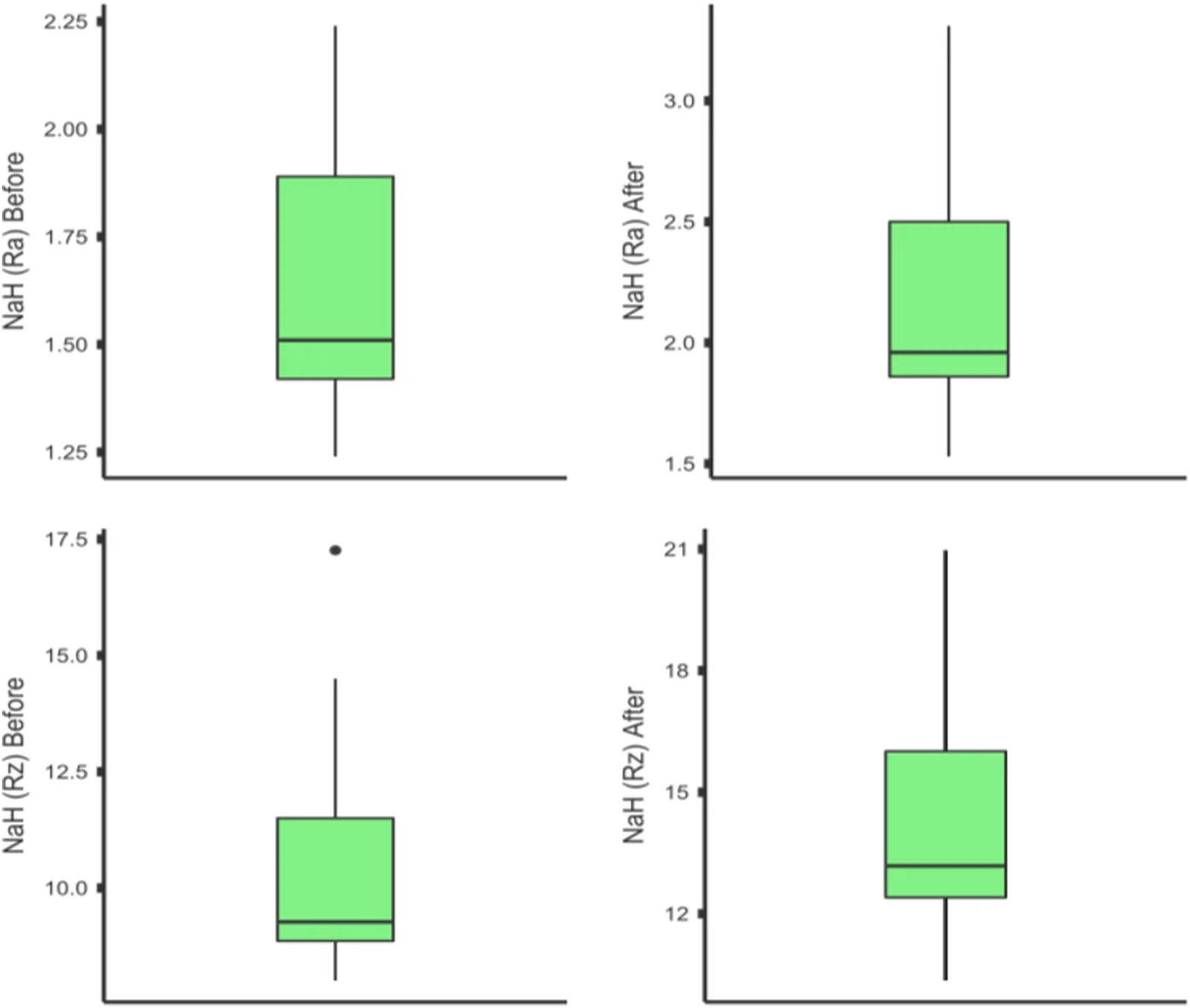
Box plot showing pre and post treatment Ra and Rz values of the discs using sodium bicarbonate.

The median Ra value of group 2 discs before treatment with glycine was 1.43 (1.40 to 1.58). After the treatment the value changed to 1.83 (1.45 to 2.33). There was a significant difference between before/after values (Table 2) with p < 0.05. The median Rz value of the untreated surfaces (a, b, c) of group 2 (glycine treated) disc was 9.30 (8.83 to 9.81). After the treatment, the median value changed to 13.26 (10.46 to 16.72) (Fig. 8). There was a statistically significant difference found in the Rz within before/after treatment with p < 0.05 (Table 2).

**Table 2:** Statistical analysis using the Wilcoxon signed rank test within the groups.

Before vs After treatment (Ra/Rz)			Statistic	p
NaHC	(Ra)	Wilcoxon W	2.00	< 0.001
	(Rz)	Wilcoxon W	8.00	< 0.001
Glycine	(Ra)	Wilcoxon W	14.50	< 0.001
	(Rz)	Wilcoxon W	5.00	< 0.001

**Fig. 8:**
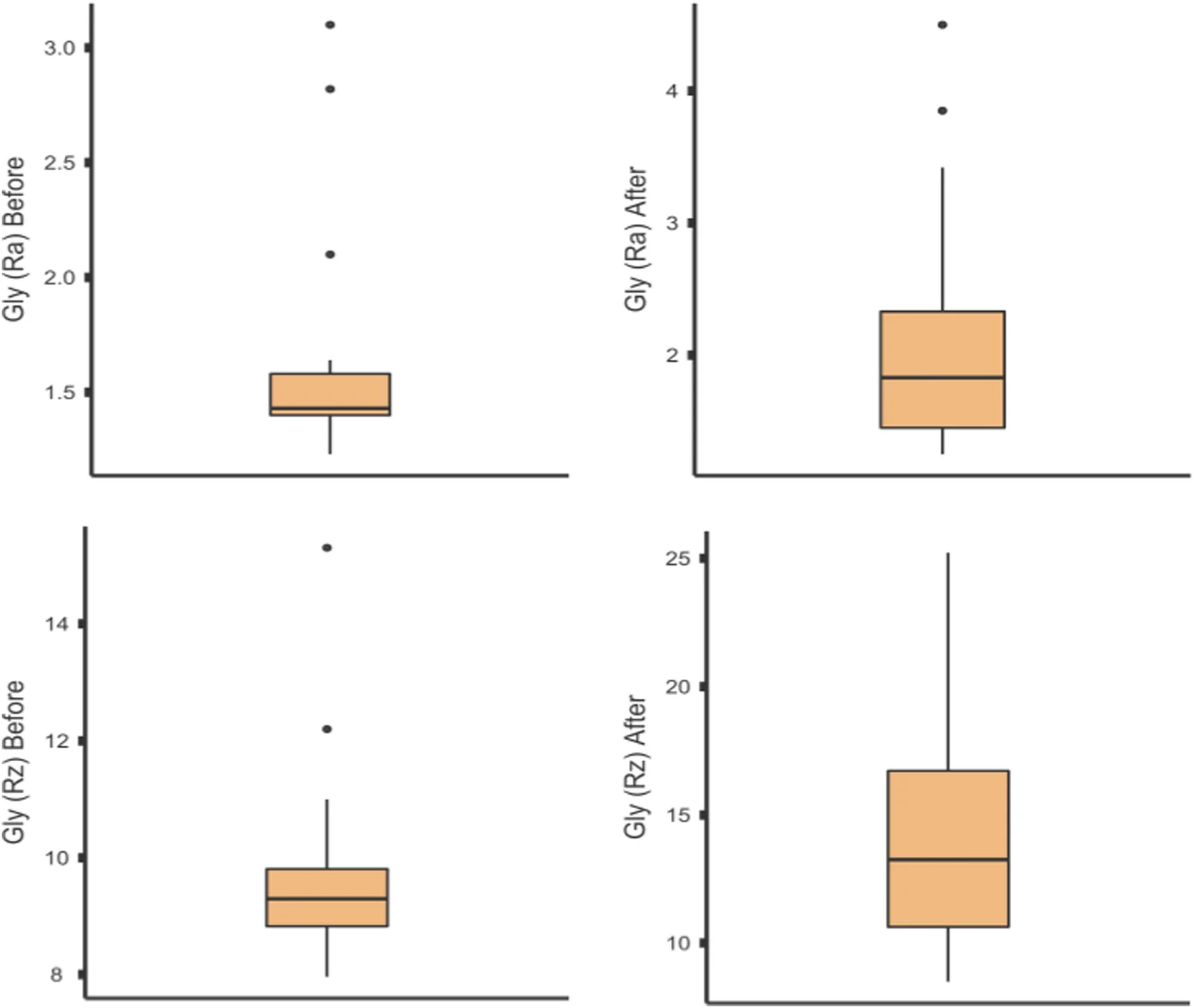
Box plot showing pre and post treatment Ra and Rz values of the discs using glycine.

Comparison of Ra and Rz values before and after treatment in group 1 and group 2 it could be inferred that the roughness of the discs increased, and the values were statistically significant.

### 6.2 Comparison across the group (Mann Whitney *U* test)

The differences in the median Ra scores before and after treatment with sodium bicarbonate and glycine were 0.480 (0.38 to 0.78) and 0.320 (0.14 to 0.54), respectively (Table 3). When the medians were compared between the groups, there was no statistically significant difference in the surface roughness post-treatment (p > 0.05) (Fig. 9a). Similarly, the Rz values also showed no significant difference between the groups using the Mann-Whitney *U* test (p > 0.05) Table 4 (Fig. 9b).

**Table 3:** Group descriptive of median values of sodium bicarbonate and glycine.

	Group	N	Median	IQR	Percentiles		
					25th	50th	75th
Ra (a,b,c) μm	NaHC	21	0.480	0.400	0.380	0.480	0.780
	Gly	21	0.320	0.400	0.140	0.320	0.540
Rz (a,b,c) μm	NaHC	21	3.810	1.940	2.780	3.810	4.720
	Gly	21	4.400	3.090	1.960	4.400	5.050

**Fig. 9:**
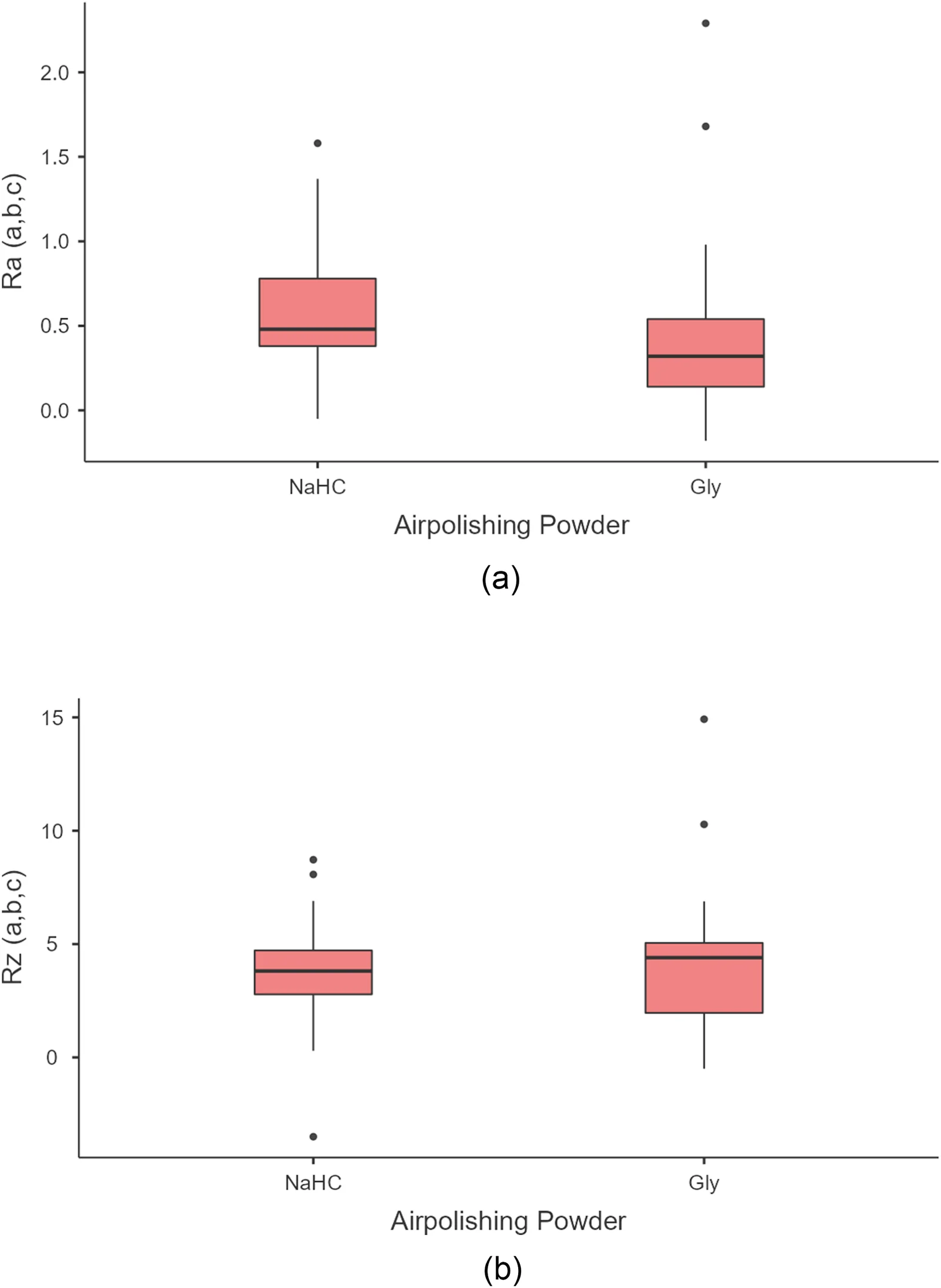
Box plot showing the comparison of post treatment Ra (a) and Rz (b) values between the groups.

**Table 4:** Statistical analysis using the Mann-Whitney *U* test between the groups.

	Statistic	P		Effect Size
Ra (a,b,c)	150	0.078	Cohen's d	0.32
Rz (a,b,c)	202	0.654	Cohen's d	0.084

The scanning electron microscopy images of sodium bicarbonate and glycine-treated discs showed an even spread of air prophy particles, and residual glycine particles were found in abundance in group 2 discs than sodium bicarbonate particles in group 1 discs. EDX analysis showed the presence of sodium (0.40%), indicative of sodium bicarbonate particles in group 1 discs (Fig. 10a), and the presence of carbon (24.89%) and nitrogen (3.41%), indicative of glycine particles in group 2 discs (Fig. 10b) (Table 5).

**Fig. 10:**
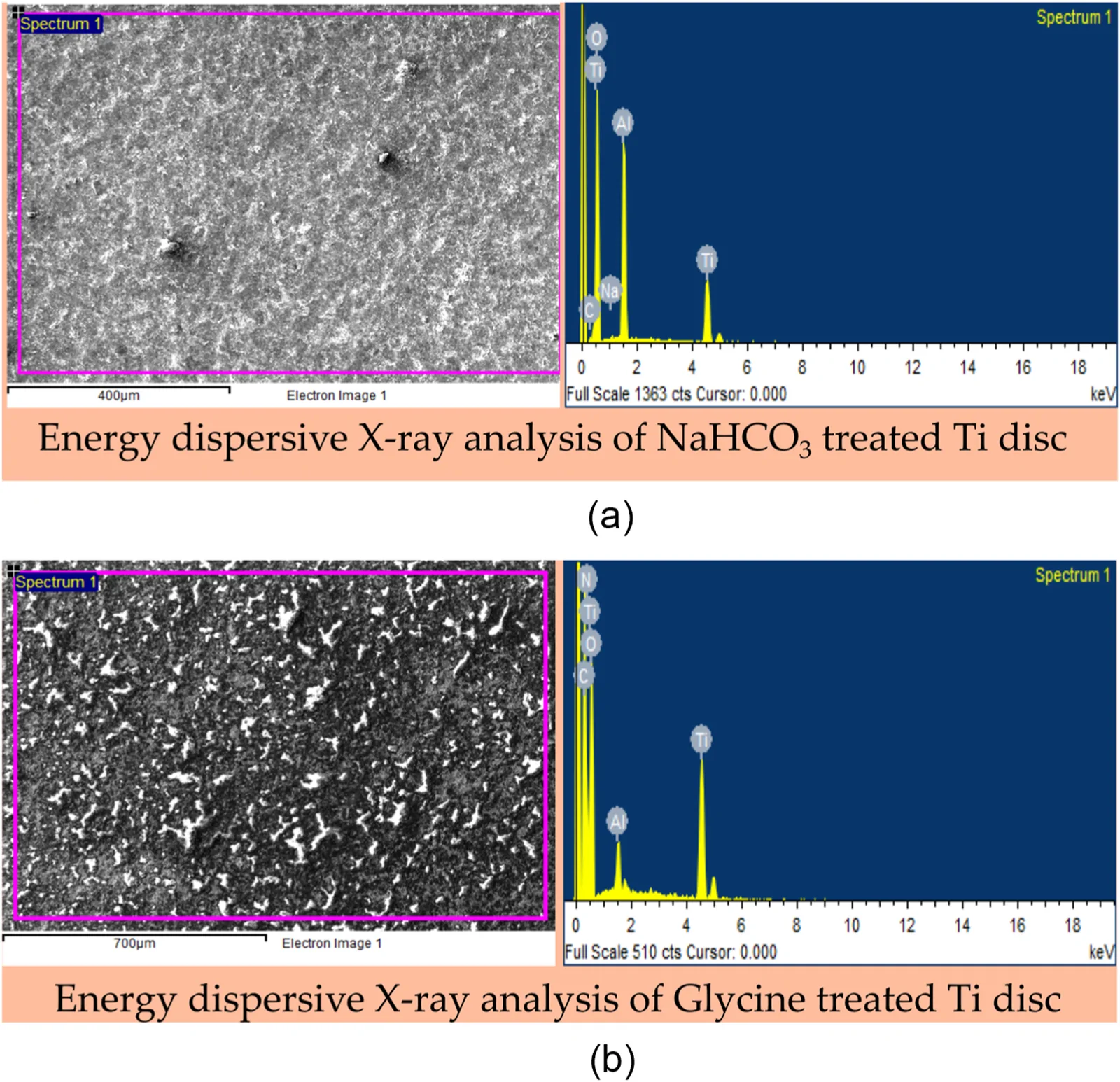
Energy Dispersive X-ray (EDX) analysis of the NaHCO_3_ (a) and Glycine (b) treated titanium discs.

**Table 5:** Showing all the elements on the surface of the titanium disc when analyzed using EDX.

Elements	Average Weight% (Sodium bicarbonate treated surface)	Average Weight% (Glycine treated surface)
C K	1.11	24.89
O K	60.3	44.46
Al K	16.82	1.65
Ti K	22.13	25.61
Na K	0.40	-
N K	-	3.41

**Total**	100%	100%

MTT assay results after 72 h of treatment with titanium discs in human gingival fibroblast (hGF) cell lines showed cell viability of 75%, 94%, 77%, 91% and 80% for 5 discs from sodium bicarbonate treated group. Cell viability was 81%, 82%, 78%, 64% and 80% for 5 discs from the glycine treated group (Fig. 11). The median values in both groups were 80% indicating no difference between the groups.

**Fig. 11:**
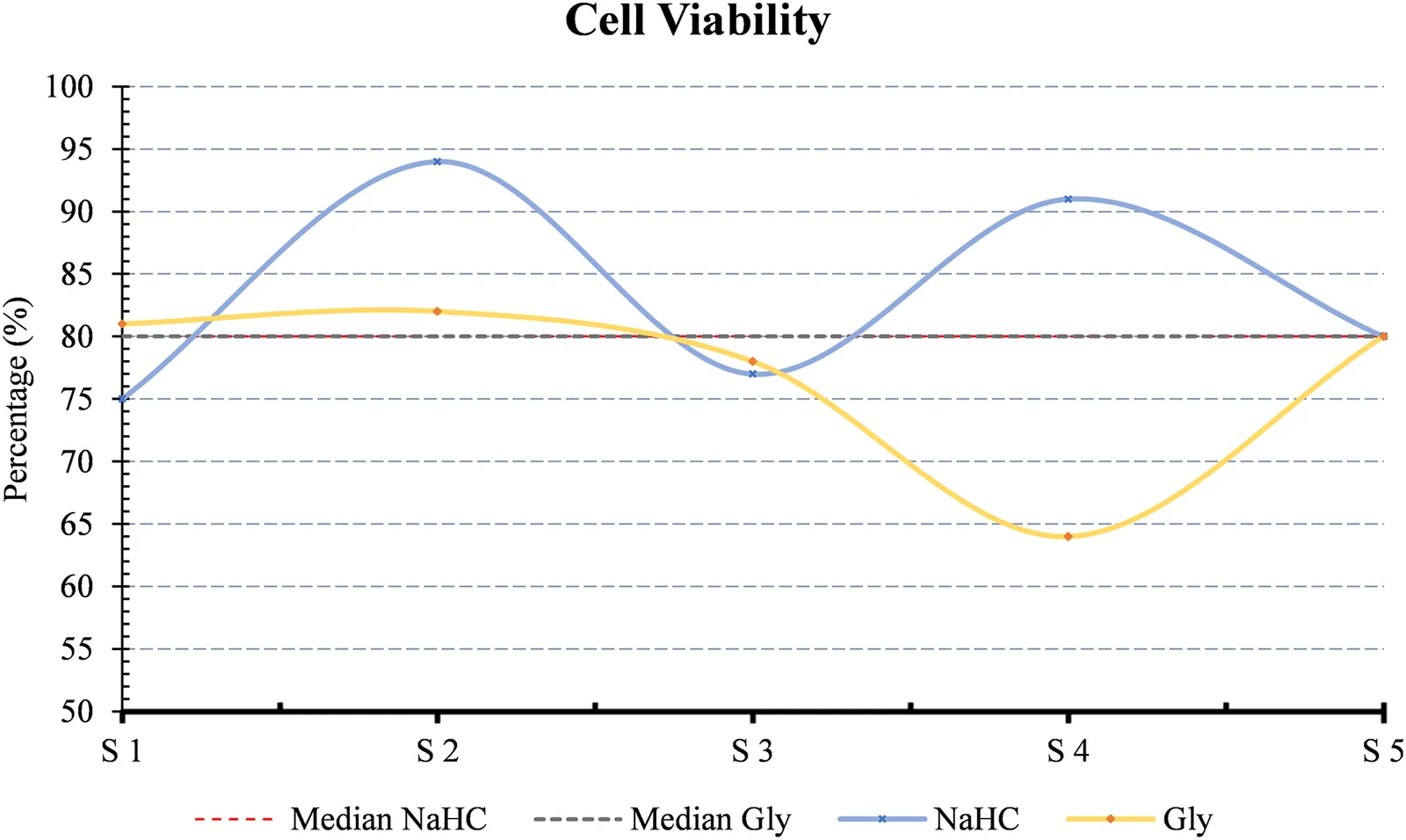
Line plot depicting the Human gingival fibroblast cell viability values of both the groups.

## 7 Discussion

In the present study, dental implant surface properties were achieved by sandblasting and acid etching of machined titanium discs. Liao et al. 2019 compared the surface properties of titanium discs after sandblasting with alumina, silicon carbide (SiC), and co-blasting with hydroxyapatite. They found that discs blasted with SiC showed increased surface roughness (Ra = 2.02 μm) and more hydrophilicity, favoring good biocompatibility of osteoblast cells.^6^ The present study reported the average depth of roughness Ra = 1.68 μm after sandblasting with silicon carbide particles and double acid etching. The variation could be due to the distance, particle size, or the duration of etching employed. To obtain the micro-roughness, double acid etching was done as per the study by Li et al. in 2012, where specimens were efficiently etched using a mixture of 18% HCl and 49% H2SO4 at 60°C for 30 min.^7^ This double acid etching process enhances bacterial antiadhesive properties (Petrini et al. 2020) and good biological properties of the titanium metal (L Giner et al. 2018).^8^^,^^9^

To simulate the hard deposits, an artificial dental calculus set (Nissin, Kyoto, Japan) was used in this study. Sergio et al. 2014 used artificial calculus with cyanoacrylate and toluidine blue 1% in equal parts to simulate natural calculus applied over titanium discs and compared four different methods for decontamination. Air powder abrasion with sodium bicarbonate completely removed artificial calculus from the surface but created some morphological alterations on the machined titanium disc surface.^2^ Motohiro et al. 2022 did a study on the decontamination of HA-coated implants, where hydroxyapatite coating was considered as calculus.^10^ Sirinirund B et al. 2019 evaluated surface topographical changes following different mechanical instrumentations on implant surfaces, where they utilized artificial calculus consisting of cyanoacrylate and toluidine blue dye.^11^ The current study applied the same type of artificial calculus consisting of silica and polymethylmethacrylate. SEM/EDX analysis showed the presence of SiO_2_ confirming the embedded artificial calculus over the discs, which both air abrasive agents effectively removed on visual inspection.

Alterations over the implant surface include both morphological and chemical changes based on the effects of each type of treatment. A study by Gehrke et al.^2^ tested the removal of hard deposits and subsequent surface changes on the titanium discs that simulated a dental implant surface after applying an air-powder abrasive system using sodium bicarbonate, which created a rough surface. The present study showed a significant difference in R_a_ values only after debriding with sodium bicarbonate. There are no published studies to date on the use of glycine air abrasive agent for the removal of artificially embedded calculus. The present study evaluated parameters similar to the research done by Gehrke et al. and compared the abrasive nature of sodium bicarbonate and glycine as well as their efficiency in debridement. A general increase in surface roughness of 1 μm after treatment shows a good depth of penetration of air abrasive agents in removing the hard deposits when directed at 45°. A previous study evaluated the implant surface alterations caused by titanium brush, air abrasive treatment using glycine powder, and diode laser.^12^ They found no significant changes in the surface roughness created over the implant surface. The results of the current study show that both the air abrasive agents caused very mild surface alterations (i.e., increased surface roughness of 1 μm), which showed a statistically significant increase within a group's before and after treatment values, but it was not statistically significant when roughness values were compared between group 1 and group 2 after treatment. The observed increase in the surface roughness in each group after treatment can be due to the type of titanium material or the amount of pressure of the air polishing powder used during the treatment procedure.^13^ The material used in our study was Grade 5 titanium, and the pressure of the air prophy unit used was 55 psi. But when the Ra and Rz values obtained after treatment of group 1 and group 2 discs were compared, no significant differences were reported, which explains the equal depth of penetration for complete removal of the hard deposits.

Even though minimally abrasive air prophy agents are used, the accumulation of residual polishing powder particles over the implant surface after decontamination affects tissue adhesion.^14^ Hence, the air abrasive agents are also required to have good biocompatibility with periodontal tissues. The analysis of surface elemental composition with the help of scanning electron microscopy and energy dispersive x-ray spectroscopy on the SLA-treated titanium discs after air abrasive treatment showed a high proportion of carbon, which indicated the presence of glycine particles. Due to some bacterial contamination and exposure to atmospheric air, the presence of carbon and nitrogen was also found. Aslihan et al. 2020 found that the element of oxygen detected on the implant surfaces can be attributable to the titanium dioxide layer which prevents the corrosion of Ti and increases biocompatibility.^15^ The TiO_2_ layer remaining on the implant surface after the treatments may be important for healing in the later stages. Lower Oxygen level was found where surface damage was seen. Thus, measurement of elemental composition in addition to SEM images provides a quantitative assessment of Ti implant surface properties. In this study, the SEM& EDX analysis showed the presence of a small amount of sodium (0.40%), indicative of sodium bicarbonate particles in group 1 discs, and the presence of carbon (34%), nitrogen (34%), indicative of a greater amount of glycine particles in group 2 discs.

The results of the MTT assay in this study showed no difference in the biocompatibility of residual sodium bicarbonate and glycine particles with human gingival fibroblast cells. This kind of reduced cell viability for amino acid glycine powder was observed by Sygkounas et al. 2017 in epithelial cells and in periodontal ligament fibroblasts when diluted 10 times.^16^ Amino acid glycine powder at its maximum soluble concentrations, showed a significant reduction in the number of human gingival fibroblasts which was attributed to the 3-4% difference in its composition from other glycine powders for which manufacturer provided no explanations.^16^ Sodium bicarbonate showed a significant decrease in the cell density as well as cell viability of human gingival fibroblasts, PDL fibroblasts, and epithelial cells with its maximum concentration.^16^ But a pronounced decrease in cell viability of osteoblast-like cells was observed for the amino acid glycine powder compared with sodium bicarbonate particles, which showed a significant increase in mitochondrial cell activity in the study by Schwarz et al., which was attributed to the larger particle size of sodium bicarbonate, which effectively eliminated oxide layers formed on the titanium surface.^17^ In turn, Seong et al. 2019 also reported a lack of cytocompatibility similar to that of pristine SLA titanium to human osteoblast cell lines with the use of glycine air abrasive powder.^18^ It was also found that the biocompatibility of sodium bicarbonate and glycine varies between non-contaminated and contaminated titanium surfaces in their application as air abrasive agents.^17^

Limitations of this study were the lack of thread design of the implant surface on the titanium discs used in this study. The assessment of attachment dynamics of artificial calculus was not done, which would have helped in simulating the natural calculus attachment on the implant surface, as well as the lack of simulation of intraoral access while using an air-powder abrasive system, which could be done using the dental implant model.

## 8 Conclusion

Both Sodium bicarbonate and Glycine were found to have no significant differences in the surface alterations. When we compared and analyzed the efficiency of these two agents in debridement, both removed artificial calculus completely within the same time. This suggests equal efficacy of both the air abrasive agents in reaching the bottom of the micro-roughened depth of the titanium disc surface, and both air abrasive powders were able to sustain up to 80% of viable human gingival fibroblast (hGF) cells.

Within the limitations of the study, it can be concluded that both air polishing powder particles are equally efficient and biocompatible in the debridement of embedded artificial calculus from SLA treated titanium discs.

## Author contribution

S.K and S.P: Study conception and design. R.M and B.R: Data Acquisition. Data was analyzed and interpreted by all authors. S.K and R.M: written the main manuscript. SUN: expert review of the final manuscript. All authors reviewed the manuscript.

## Ethics approval and consent to participate

Ethical approval was obtained from Kasturba hospital (KMC and KH). The ethical clearance number is IEC:808/2020. Informed consent is not applicable.

## Funding

No funding was obtained for this study.

## Declaration of competing interest

All authors declare that they have no known competing financial interests or personal relationships that could have appeared to influence the work reported in this paper.
